# Supplementary material for: Genotype networks of 80 quantitative Arabidopsis thaliana phenotypes reveal phenotypic evolvability despite pervasive epistasis
Source: PLoS Comput Biol. 2020 Aug 13;16(8):e1008082. doi: 10.1371/journal.pcbi.1008082 (PMC7447023; doi:10.1371/journal.pcbi.1008082)
Supplement: S1 Text — (DOCX) [file pcbi.1008082.s001.docx]

***S1 Text: Characteristics of genotype networks based on random nucleotide strings***

To evaluate if properties of the four example genotype networks illustrated in Fig 2 would be expected to occur by chance alone for data sets of similar size and genetic diversity, we created 10,000 random genotype networks for each of the four phenotypes, computed network descriptors for them, and compared these descriptors with their values in the four biological networks. We built these 10,000 random networks from 10,000 sets of randomized genotype strings with the same nucleotide composition as the biologically observed genotypes (see Methods). The number of unique genotype sequences that this randomization procedure yielded was consistently higher than in the biological data for all 10,000 sets of strings and for all four phenotype categories (S3A Fig). In other words, the biologically observed genotype sequences are less diverse than expected by chance. One possible reason is that the corresponding genomic positions are under selection, because they are strongly associated with the investigated phenotype and thus functionally important. The number of squares in the genotype networks based on biological data lay within the range of the number of squares in the random genotype networks, with the exception of the defense phenotype bacterial growth, where the genotype network based on biological data showed a higher number of squares (S3B Fig). Moreover, the size of the largest network component of the flowering phenotype plant diameter at flowering and the defense phenotype bacterial growth lay within the range of the values obtained from random networks. In contrast, the largest component size was lower in the biological networks compared to random networks for the ion phenotype arsenic concentration and the developmental phenotype plant width (S3C Fig). We also determined the maximum vertex betweenness, that is, the highest number of shortest path that pass through a vertex (S3D Fig). It showed that the vertex betweenness of genotype networks based on biological data is in the lower range of those values obtained from random networks. For two phenotypes – the ion phenotype arsenic concentration and the development phenotype plant width – vertex betweenness was lower than in the random genotype networks, showing that vertices with very high betweenness can occur in random genotype networks of the size we study here.
