# Supplementary material for: Genotype networks of 80 quantitative Arabidopsis thaliana phenotypes reveal phenotypic evolvability despite pervasive epistasis
Source: PLoS Comput Biol. 2020 Aug 13;16(8):e1008082. doi: 10.1371/journal.pcbi.1008082 (PMC7447023; doi:10.1371/journal.pcbi.1008082)
Supplement: S2 Text — (DOCX) [file pcbi.1008082.s002.docx]

***S2 Text: Mutational path length is weakly correlated to phylogenetic distance***

The greater the distance is between two vertices in a genotype network, the greater is the number of mutations at phenotype-associated genomic positions that are needed to reach one vertex genotype from the other. We refer to this number of mutations as mutational path length. This length is not necessarily related to the phylogenetic distance between two accessions, because two accessions might be phylogenetically very different yet have very similar genotypes at the positions determining any one phenotype, indicating conservation of that genotype. Conversely, they may be phylogenetically very closely related, but have diverged greatly at the genomic positions affecting a focal phenotype, possibly indicating rapid evolution of the phenotype. To characterize the relationship between phylogenetic distance and mutational path length, we constructed a phylogenetic tree based on all 214,051 polymorphic loci of the 199 *A. thaliana* accessions used by Atwell and colleagues (2010). We used two measures of phylogenetic distances, namely the number of internal nodes (branch-points) that separate two accessions on the phylogenetic tree (S4A Fig), and the pairwise nucleotide distance between two accessions. For both measures, we found only weak correlations between the length of the shortest path between two accessions in a genotype network and phylogenetic distance in the four example phenotypes plant diameter at flowering, arsenic concentrations, bacterial growth, and plant width (S4B Fig and S4D Fig). More generally, mutational path length and the number of internal nodes (number of nucleotide differences) were weakly correlated for 26 (28) phenotypes (S4C Fig and S4E Fig, respectively). For the remaining phenotypes, we could not detect significant correlations.

Below, we discuss examples for two classes of noteworthy cases. First, two accessions may be neighbors in a genotype network (that is, they differ by one nucleotide in genomic positions that affect a phenotype), but they may nonetheless be phylogenetically distantly related, as quantified by the number of internal nodes or nucleotide differences between them. Second, two accessions may be closely related in phylogenetic terms, but separated by a long mutational path in a genotype network. Examples for the first scenario comprise the phenotypes sodium and molybdenum concentrations (phenotype 5 and 73) as well as leaf necrosis (phenotype 23). A single edge in the genotype networks of these three phenotypes connected one pair of accessions that are single-mutant neighbors, but that are phylogenetically separated by 27, 25.3 and 24.8 internal nodes, respectively (across all pairwise comparisons, the maximum number of internal nodes separating two accessions was 28). When using the number of nucleotide differences as a phylogenetic measure, we found that the genotype networks of the phenotypes days to germination at 10°C (phenotype 59), bacterial disease resistance (phenotype 70), and leaf number when the bolt reached 5 cm (phenotype 36) contained a single edge that connects two accessions as one-mutant neighbors, but the pair of accessions constituting this edge differed by 86,054 nucleotides, 85875.6 nucleotides, and 84,831.05 nucleotides, respectively. The maximum number of nucleotide differences between all pairs of accessions is 94,561.

Examples for the second scenario (close phylogenetic relations, but long mutational path between accessions) include the phenotype leaf number at flowering time (phenotype 41), plant width at 16°C (phenotype 14), and number of days between appearance of the first flower and senescence of the last flower (phenotype 32). The genotype network of these phenotypes contained accessions that are separated by five or more mutational steps in the genotype network, but only by one internal node in the phylogenetic tree.
