# Supplementary material for: Genotype networks of 80 quantitative Arabidopsis thaliana phenotypes reveal phenotypic evolvability despite pervasive epistasis
Source: PLoS Comput Biol. 2020 Aug 13;16(8):e1008082. doi: 10.1371/journal.pcbi.1008082 (PMC7447023; doi:10.1371/journal.pcbi.1008082)
Supplement: S3 Text — (DOCX) [file pcbi.1008082.s003.docx]

***S3 Text: Effect of potential linkage disequilibrium on epistasis and path accessibility***

One challenge in the analysis of GWAS data is the difficulty to discriminate between ‘causal’ genomic positions (loci) – those that affect a phenotype directly – and genomic positions that are only in high linkage disequilibrium (LD) with causal positions (Korte and Farlow, 2013). Atwell and colleagues (2010) reported that LD is usually too widespread to directly identify causal positions with a GWAS alone. We wanted to find out whether LD substantially affects the incidence of epistasis and the fraction of accessible mutational paths to maximum phenotypic values. Previous studies reported different degrees of LD in *A. thaliana*, and estimated LD to decay within 250 kbp (Nordborg *et al*., 2002), 10 kbp (Kim *et al*., 2007), and 5 kbp (Gan *et al*., 2011). The difference between these results originates from different methods to estimate LD, and from using global or local *A. thaliana* populations. To be conservative in our analysis, we assumed that any two genomic positions that are less than 250kbp apart might be in LD. We screened the genotype strings that we used to build genotype networks (at *P*-values that yield the largest number of squares) for loci within 250 kbp of each other. We found such positions in the genomic data for 60 phenotypes (S6 Table). Depending on the phenotype, we identified between zero and seven groups of genomic positions within 250 kbp of each other, and each group comprised between two and 11 positions (S6 Table). We then resampled genotype strings by choosing only one representative genomic position from each LD group, and did so for all possible combinations of positions. For example, if we found two LD groups comprising two and three genomic positions within a 250kbp window, respectively, we reconstructed 2×3=6 resampled nucleotide strings for this phenotype. This procedure resulted in between 2 and 288 different nucleotide strings for each phenotype, which we used to reconstruct genotype networks. We then calculated the frequency of epistasis and the fraction of accessible mutational paths for these genotype networks. Our observations are summarized in S6 Fig, which shows that epistasis is still pervasive, and that mutational paths still remain accessible. In summary, eliminating closely linked genomic position that may show LD from our analysis, does not affect our conclusions.

Cited References

Atwell S, Huang YS, Vilhjalmsson BJ, Willems G, Horton M, Li Y, Meng D, Platt A, Tarone AM, Hu TT, Jiang R, Muliyati NW, Zhang X, Amer MA, Baxter I, Brachi B, Chory J, Dean C, Debieu M, de Meaux J, Ecker JR, Faure N, Kniskern JM, Jones JD, Michael T, Nemri A, Roux F, Salt DE, Tang C, Todesco M, Traw MB, Weigel D, Marjoram P, Borevitz JO, Bergelson J, Nordborg M (2010) Genome-wide association study of 107 phenotypes in Arabidopsis thaliana inbred lines. *Nature* 465, 627-631.

Gan X, Stegle O, Behr J, Steffen JG, Drewe P, Hildebrand KL, Lyngsoe R, Schultheiss SJ, Osborne EJ, Sreedharan VT, Kahles A, Bohnert R, Jean G, Derwent P, Kersey P, Belfield EJ, Harberd NP, Kemen E, Toomajian C, Kover PX, Clark RM, Rätsch G, Mott R (2011) *Nature* 477, 419-423.

Kim S, Plagnol V, Hu TT, Toomajian C, Clark RM, Ossowski S, Ecker JR, Weigel D, Nordborg M (2007) Recombination and linkage disequilibrium in Arabidopsis thaliana. *Nature Genetics* 39(9), 1151-1155.

Korte A, Farlow A (2013) The advantages and limitations of trait analysis with GWAS: a review. *Plant Methods* 9, article 29.

Nordborg M, Borevitz JO, Bergelson J, Berry CC, Chory J, Hagenblad J, Kreitman M, Maloof JN, Noyes T, Oefner PJ, Stahl EA, Weigel D (2002) The extent of linkage disequilibrium in Arabidopsis thaliana. *Nature Genetics* 30(2), 190-193.
