# Supplementary material for: Genotype networks of 80 quantitative Arabidopsis thaliana phenotypes reveal phenotypic evolvability despite pervasive epistasis
Source: PLoS Comput Biol. 2020 Aug 13;16(8):e1008082. doi: 10.1371/journal.pcbi.1008082 (PMC7447023; doi:10.1371/journal.pcbi.1008082)
Supplement: S4 Text — (DOCX) [file pcbi.1008082.s004.docx]

***S4 Text: Structure of the phylogenetic tree of all 199 A. thaliana accessions in Newick format***

(((((6965:0.11947,8231:0.16595)1.000:0.07832,(6964:0.18878,(8242:0.14674,(7519:0.13946,7518:0.15036)1.000:0.05146)1.000:0.04362)1.000:0.03232)1.000:0.03791,(8230:0.23414,((6905:0.23630,(6921:0.00900,6920:0.00722)1.000:0.21734)1.000:0.07578,((9058:0.19397,(8351:0.18740,((6969:0.01934,6968:0.01276)1.000:0.19509,(((6016:0.11244,6064:0.08910)1.000:0.03657,((6918:0.06291,6917:0.06659)1.000:0.07415,(6913:0.08372,6009:0.05839)1.000:0.06074)1.000:0.02762)1.000:0.04637,(8376:0.14489,((6043:0.01075,6046:0.01201)1.000:0.11112,(6900:0.01097,6901:0.00903)1.000:0.11060)1.000:0.05878)1.000:0.02955)1.000:0.03646)1.000:0.03399)1.000:0.05312)1.000:0.03410,((7516:0.07581,7517:0.06367)1.000:0.11888,6074:0.20963)1.000:0.04069)0.991:0.01976)1.000:0.02623)1.000:0.02508)1.000:0.01468,(((8249:0.18284,8222:0.18690)1.000:0.04555,(6974:0.19817,(8241:0.16725,8326:0.18629)0.981:0.03083)1.000:0.02005)1.000:0.02806,((6932:0.21420,7346:0.17669)1.000:0.04086,8283:0.22155)1.000:0.02786)0.702:0.01700)1.000:0.01398,(((8306:0.16102,(8423:0.14408,8426:0.15765)1.000:0.04764)1.000:0.05676,(8240:0.21050,(8237:0.16152,(8422:0.01677,8266:0.03585)1.000:0.13268)1.000:0.04415)1.000:0.03377)1.000:0.03048,((6946:0.21058,(8256:0.15720,(8258:0.01371,8259:0.01639)1.000:0.14409)1.000:0.07552)1.000:0.03083,(9057:0.20142,((8369:0.09961,(6040:0.06552,8335:0.05301)1.000:0.03632)1.000:0.06621,(6973:0.19563,8247:0.06266)1.000:0.07966)1.000:0.06442)1.000:0.04795)0.991:0.01567)1.000:0.01814,((((((6976:0.14105,6975:0.13967)1.000:0.06159,(8311:0.20332,8314:0.24478)1.000:0.04596)1.000:0.02297,((((((7460:0.15684,(6984:0.13670,8285:0.14816)1.000:0.03621)0.000:0.01868,(8236:0.18716,8412:0.17107)1.000:0.04178)0.911:0.01573,((8284:0.13586,6008:0.14782)1.000:0.04430,(6903:0.19826,(6985:0.14482,5837:0.15385)1.000:0.03647)0.709:0.01697)1.000:0.02149)1.000:0.02115,((7520:0.14893,7521:0.15200)1.000:0.05502,((8300:0.15512,7296:0.17518)1.000:0.04614,(8378:0.18096,(8334:0.21031,8389:0.14127)0.954:0.03168)1.000:0.02091)0.949:0.01256)1.000:0.02139)1.000:0.01661,((8365:0.15800,7424:0.16439)1.000:0.04880,((6951:0.15981,(6956:0.07838,6957:0.06408)1.000:0.09396)1.000:0.03777,(8235:0.17347,8313:0.17866)1.000:0.04280)1.000:0.01679)1.000:0.01604)0.299:0.01644,(7275:0.18838,6243:0.18354)1.000:0.04893)1.000:0.01914)1.000:0.02046,(((7163:0.23357,100000:0.16726)1.000:0.08911,((6978:0.16994,8325:0.18957)1.000:0.03618,((8354:0.14909,(7438:0.14663,(7323:0.12776,((8424:0.09970,(6962:0.06085,(6963:0.07289,6929:0.04555)1.000:0.02373)1.000:0.03178)1.000:0.05498,(6931:0.10523,6930:0.10847)1.000:0.04124)0.999:0.02225)1.000:0.03316)1.000:0.05039)1.000:0.05134,6981:0.20754)1.000:0.02909)0.964:0.01902)1.000:0.02494,(6980:0.19604,(6919:0.19334,(8388:0.13210,(8290:0.01137,6910:0.00990)1.000:0.11193)1.000:0.06397)1.000:0.03057)1.000:0.02748)1.000:0.01910)1.000:0.02168,(6042:0.22583,(7477:0.20011,(6899:0.22983,(6972:0.01069,8395:0.00700)1.000:0.21031)0.975:0.04214)1.000:0.03121)1.000:0.02987)1.000:0.02518,(((((8343:0.20087,8270:0.15841)0.262:0.03910,6937:0.19158)1.000:0.02735,(6979:0.17373,7418:0.17832)1.000:0.04764)1.000:0.03675,((((8275:0.20273,(6977:0.19925,(6936:0.13298,6958:0.12347)1.000:0.07205)1.000:0.03337)1.000:0.04378,(((6959:0.13101,6960:0.12936)1.000:0.08989,(8329:0.17753,8213:0.18729)1.000:0.04352)1.000:0.02234,((((6924:0.15357,(6943:0.14815,((6966:0.08944,8254:0.05956)1.000:0.05280,(6944:0.09149,(7064:0.11884,6923:0.08907)1.000:0.02712)0.923:0.02009)1.000:0.05672)1.000:0.03757)1.000:0.04921,(8214:0.16623,(6709:0.02632,6908:0.00935)1.000:0.14562)1.000:0.03796)1.000:0.02758,((8215:0.16399,7306:0.19057)1.000:0.04927,(8245:0.01434,6926:0.01360)1.000:0.17812)1.000:0.02236)1.000:0.02665,8297:0.22455)1.000:0.02116)1.000:0.02542)1.000:0.01551,((6928:0.18188,(7523:0.18381,(6983:0.12115,((((8233:0.01042,7033:0.01003)1.000:0.00207,(6927:0.00884,7515:0.00838)1.000:0.00192)0.072:0.00149,7526:0.00729)1.000:0.03018,(7524:0.01278,7525:0.18466)1.000:0.05293)1.000:0.04924)1.000:0.04552)1.000:0.08863)1.000:0.06388,((6967:0.16390,6907:0.16951)1.000:0.05746,(6914:0.20681,7514:0.23933)1.000:0.04160)1.000:0.02656)1.000:0.02958)1.000:0.01785,(((8337:0.16086,(6904:0.17825,8243:0.14403)1.000:0.03995)1.000:0.05314,(6897:0.21090,(6906:0.18997,8353:0.16062)1.000:0.06259)0.174:0.02061)1.000:0.02107,((8264:0.18676,((6961:0.12867,8357:0.12810)1.000:0.04161,(6971:0.14387,(6970:0.11647,6933:0.11548)1.000:0.05097)1.000:0.02607)1.000:0.03050)1.000:0.06702,((7522:0.20472,(8274:0.16016,6911:0.19308)0.489:0.05560)1.000:0.06061,(6988:0.18099,7081:0.16653)1.000:0.05313)1.000:0.02916)1.000:0.03863)1.000:0.01676)1.000:0.01813)1.000:0.02166,(((((7062:0.16342,7014:0.15879)1.000:0.07102,((8387:0.06028,6915:0.04404)1.000:0.14946,6982:0.20061)1.000:0.02664)1.000:0.02885,((8374:0.20226,7231:0.19056)0.812:0.03727,8239:0.25334)1.000:0.02703)1.000:0.02206,(((8312:0.21008,(8420:0.18422,7094:0.17982)1.000:0.04050)1.000:0.03499,((6940:0.17601,6942:0.19329)1.000:0.04298,((8411:0.00378,(8366:0.00798,6916:0.01542)0.568:0.00310)1.000:0.23309,(7000:0.20400,8271:0.19677)1.000:0.04001)1.000:0.02969)1.000:0.02426)1.000:0.01613,((8323:0.16721,6898:0.20127)1.000:0.07282,((7255:0.21219,(8296:0.22045,6939:0.17197)1.000:0.03889)1.000:0.01822,((6922:0.16277,(6909:0.12794,7461:0.09343)1.000:0.17047)1.000:0.04662,(7282:0.17849,7123:0.18348)1.000:0.03170)1.000:0.02452)1.000:0.03398)1.000:0.02199)1.000:0.01900)1.000:0.02303,((8310:0.19672,8265:0.20580)1.000:0.04651,(7147:0.21169,(6945:0.17630,8430:0.18666)1.000:0.05292)1.000:0.02744)1.000:0.03546)1.000:0.01914)1.000:0.02393)1.000:0.01878);
