# Supplementary figures and images for: Genotype networks of 80 quantitative Arabidopsis thaliana phenotypes reveal phenotypic evolvability despite pervasive epistasis
Source: PLoS Comput Biol. 2020 Aug 13;16(8):e1008082. doi: 10.1371/journal.pcbi.1008082 (PMC7447023; doi:10.1371/journal.pcbi.1008082)

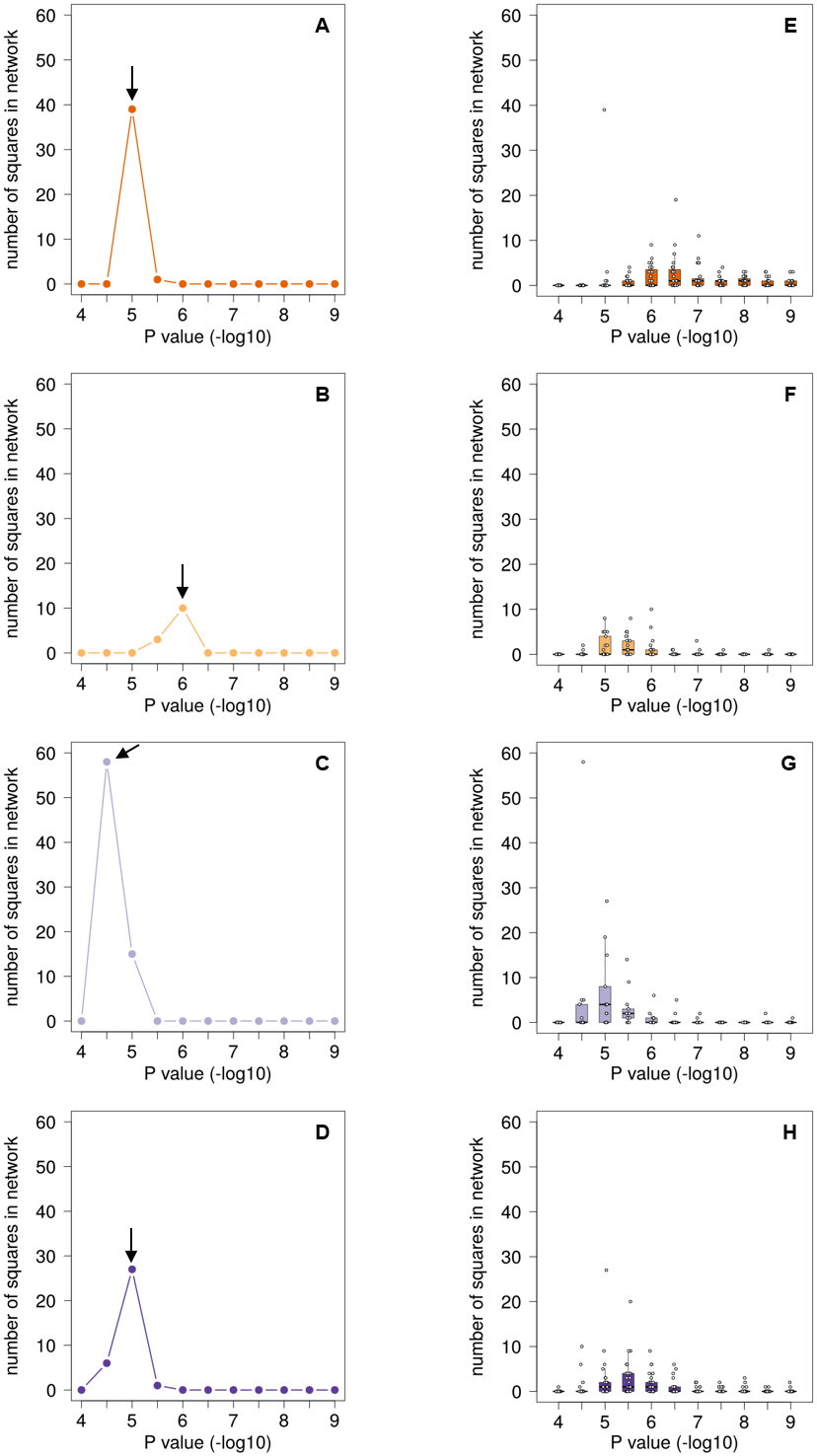

Supplement: S1 Fig — We asked which P-value cutoff between 10-4 and 10-9 (horizontal axes) results in the largest number of squares (vertical axes) for each of our 80 phenotypes. (A – D) For each of the four example phenotypes discussed in the main text, one single P-value cutoff leads to the largest number of squares. This P-value cutoff was 10-5 for the phenotype leaf diameter at flowering (A), 10-6 for the phenotype arsenic concentration (B), 10-4.5 for the phenotype bacterial growth (C), and 10-5 for the phenotype plant width (D). Arrows in each panel indicate the P-value that yields the largest number of squares in each of the four phenotype categories. (E – H) Box plots show the distribution of square number for all phenotypes in the categories flowering (E), ions (F), defense (G), and development (H). In each box plot, the central horizontal line indicates the median value, the lower box limit shows the value of the first quartile, the upper box limit depicts the third quartile, the whiskers indicate values within the 1.5-fold interquartile range, and each open circle represents one data point. (TIF) [file pcbi.1008082.s011.tif]

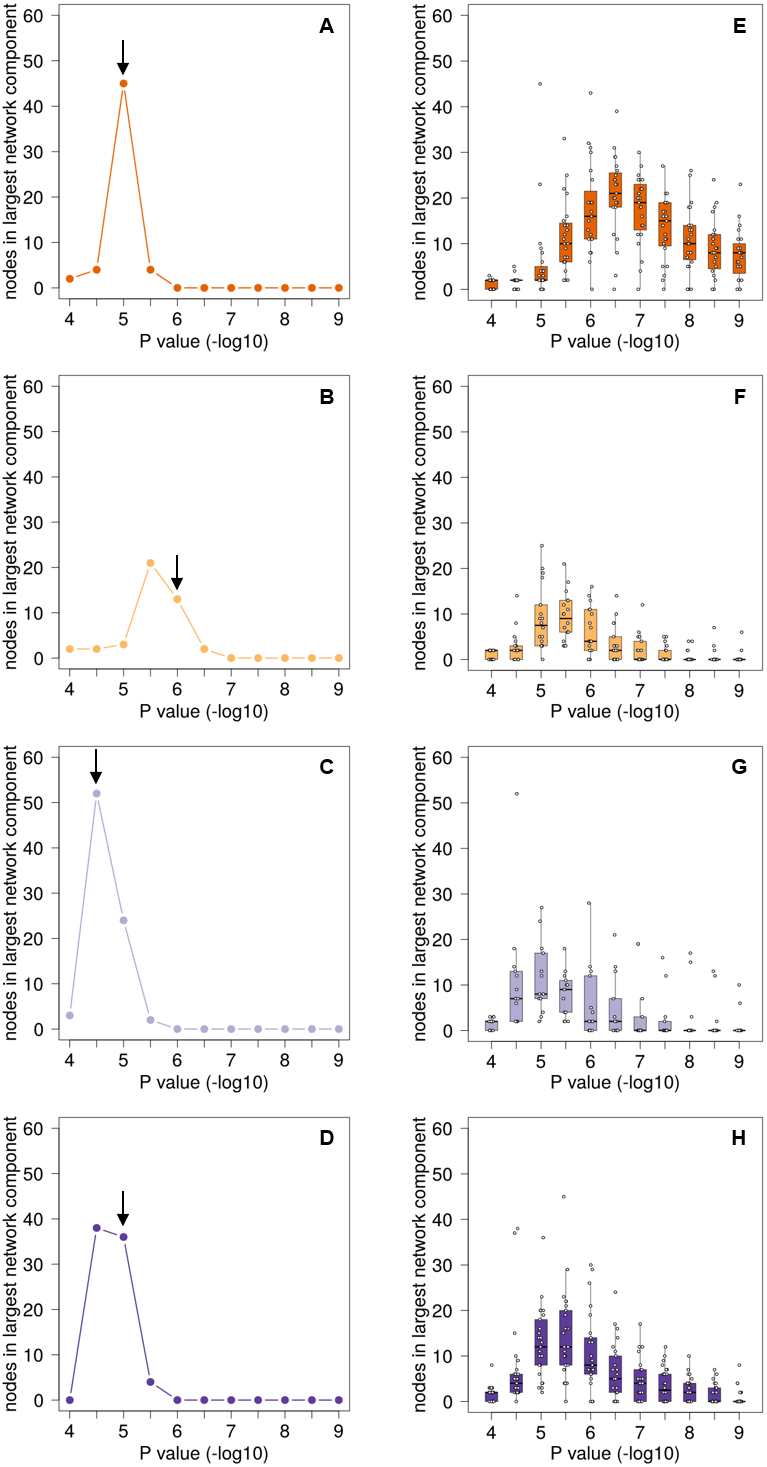

Supplement: S2 Fig — We asked which P-value cutoff between 10-4 and 10-9 (horizontal axes) results in the largest connected component (vertical axes), and did so for each of our 80 phenotypes. Panels A to D show the results of the four example phenotypes discussed in the main text, namely leaf diameter at flowering (A), arsenic concentration (B) bacterial growth (C), and plant width (D). Arrows in each panel indicate the P-value that was chosen based on the number of squares in the genotype network, as shown in S1 Fig. Panels E to H show box plots with the distribution of the sizes of largest network components for all phenotypes in the categories flowering (E), ions (F), defense (G), and development (H). In each box plot, the central horizontal line indicates the median value, the lower box limit shows the value of the first quartile, the upper box limit depicts the third quartile, the whiskers indicate values within the 1.5-fold interquartile range, and each open circle shows one data point. (TIF) [file pcbi.1008082.s012.tif]

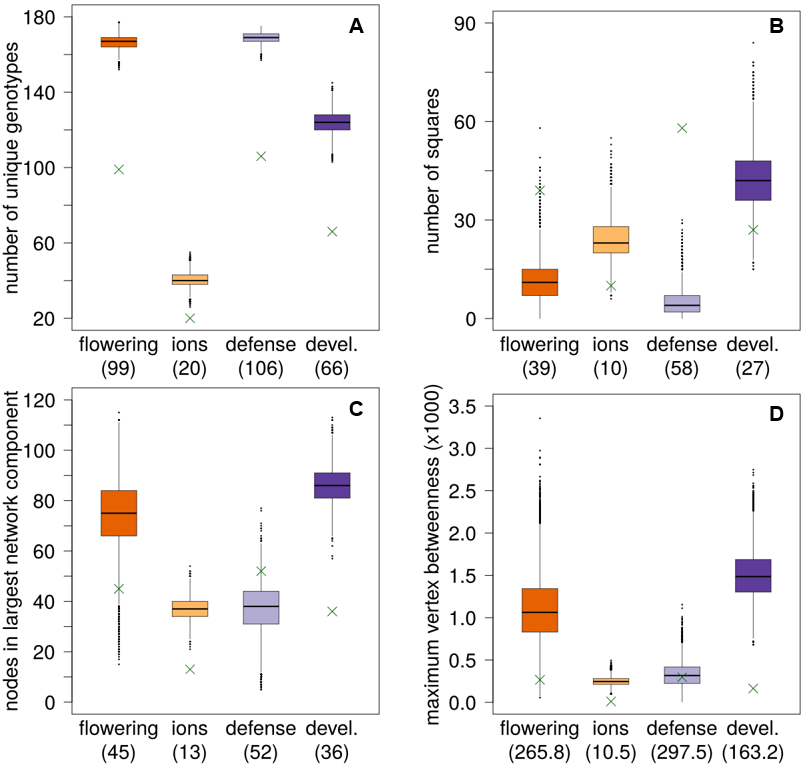

Supplement: S3 Fig — We computed the number of unique genotypes (A), the number of squares in a genotype network (B), the number of vertices in the largest component with a size of at least two (C), and the maximum value of vertex betweenness (D) resulting from 10,000 randomly generated genotype networks. Box plots illustrate results from 10,000 replicates, and do so separately for each phenotype category (“devel”: development). Green crosses and numbers in parentheses on the horizontal axis indicate the empirically observed values for the example phenotypes in the flowering (plant diameter at flowering), ions (arsenic concentration), defense (bacterial growth) and development (“devel”, plant width) categories. (TIF) [file pcbi.1008082.s013.tif]

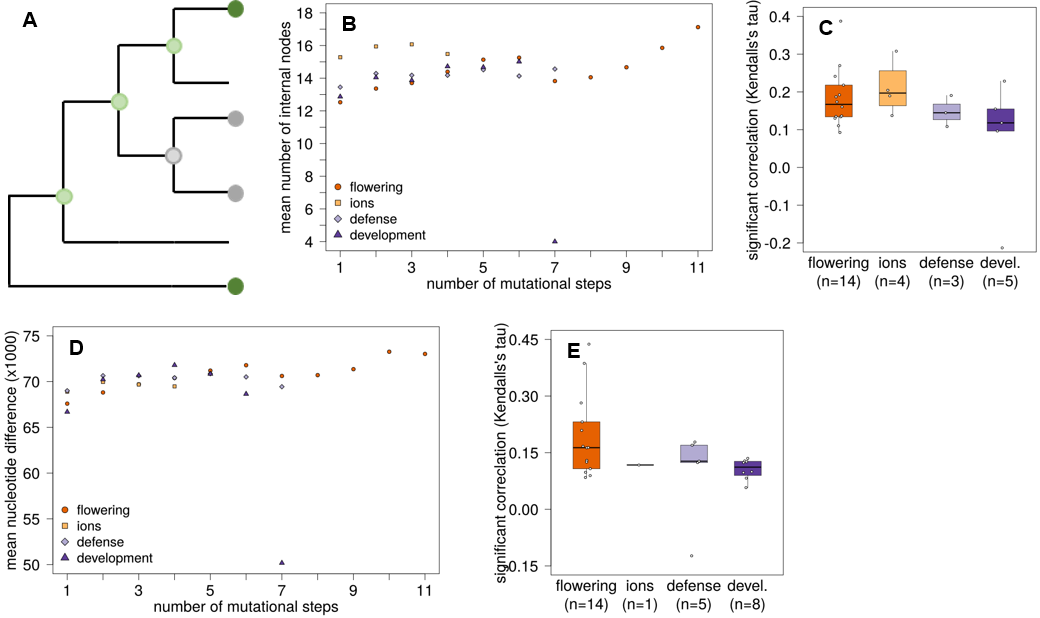

Supplement: S4 Fig — We compared the mutational path length between two genotypes (accessions) in our genotype networks with the number of branch-points separating these accessions on a phylogenetic tree. (A) Principle of counting internal branch-points. An accession (vertex) in the genotype network corresponds to one leaf on the phylogeny tree shown in S7 Fig. For example, the two leaves shown here as dark grey circles are separated by one internal branch-point (light grey circle), and the two leaves shown as dark green circles are three internal branch-points apart (light green circles). (B) For our four example genotype networks shown in Fig 2, we estimated the length of the shortest path between all vertices. We then sorted all path according to their length (horizontal axis) and calculated the mean number of internal branch-points between two accessions separated by a given path length (vertical axis). We did this individually for the phenotype plant diameter at flowering (dark orange circles), zinc concentration (light orange squares), bacterial growth (light purple diamonds), and plant width (dark purple triangles). None of the four phenotypes showed a strong association between the number of internal branch points and the length of a mutational path (Kendall’s tau between -0.021 and 0.388; P-values ≤ 0.0473). (C) We extended the same analysis to all 80 phenotypes. Each box plot summarizes rank correlation coefficients (Kendall’s tau) for one phenotype category (devl, development). In each box plot, the central horizontal line indicates the median value, lower and upper box limits show the first and third quartile, whiskers indicate values within the 1.5-fold interquartile range, and each open circle shows one data point. The number n of phenotypes in each category with correlations significantly different from zero is shown on the horizontal axes. Panels (D) and (E) are analogous to panels (B) and (C), but show the number of nucleotide differences between two accessions as a phy [file pcbi.1008082.s014.tif]

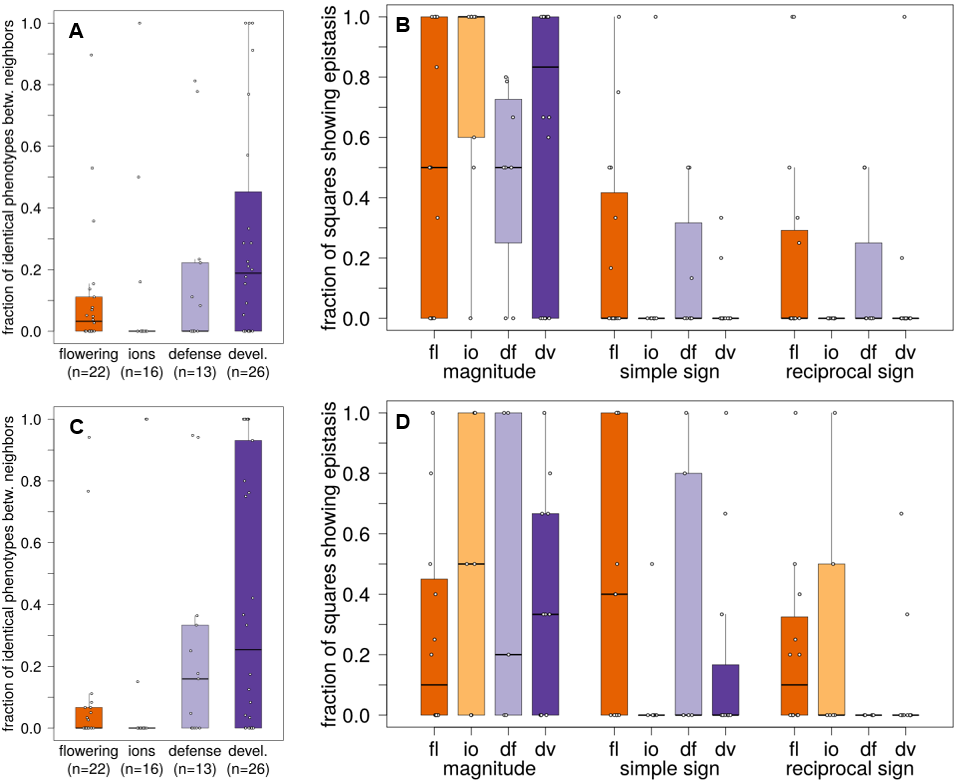

Supplement: S5 Fig — To determine how sensitive our quantification of epistasis is to the chosen P-value cutoff, we created genotype networks with non-optimal P-values for all phenotypes (i.e. P-values that do not yield the largest number of squares in a genotype network). Specifically, we used P-values whose -log10 value is higher (panels A and B) or lower (panels C and D) by 0.5 compared to the individual optimal P-value of each phenotype. (A and C) Fraction of genotype network neighbors with identical phenotypic values as a result of higher (panel A) or lower (panel C) P-values. We consider the absolute difference between the phenotypic values of two neighbors as identical if it does not exceed the coefficient of phenotypic variation (δ) for accessions with the same genotype at all phenotype-associated loci (Methods). We plot the fraction of neighbors with identical phenotypic values (vertical axis) in the form of a box plot for each of the four phenotype categories (“devl.”: development). The number of phenotypes in each category is shown on the horizontal axis as sample size (n). (B and D) Frequency of different types of epistasis for higher (panel B) or lower (panel D) P-values to reconstruct genotype networks. The vertical axis shows the fraction of squares with magnitude, simple sign, and reciprocal sign epistasis as a box plot, where data are grouped according to the type of epistasis and phenotype category (horizontal axis; “fl”: flowering; “io”: ions; “df”: defense; “dv”: development). In each box plot, the central horizontal line indicates the median value, lower and upper box limit shows the values of the first and third quartile, whiskers indicate values within the 1.5-fold interquartile range, and each open circle shows one data point. (TIF) [file pcbi.1008082.s015.tif]

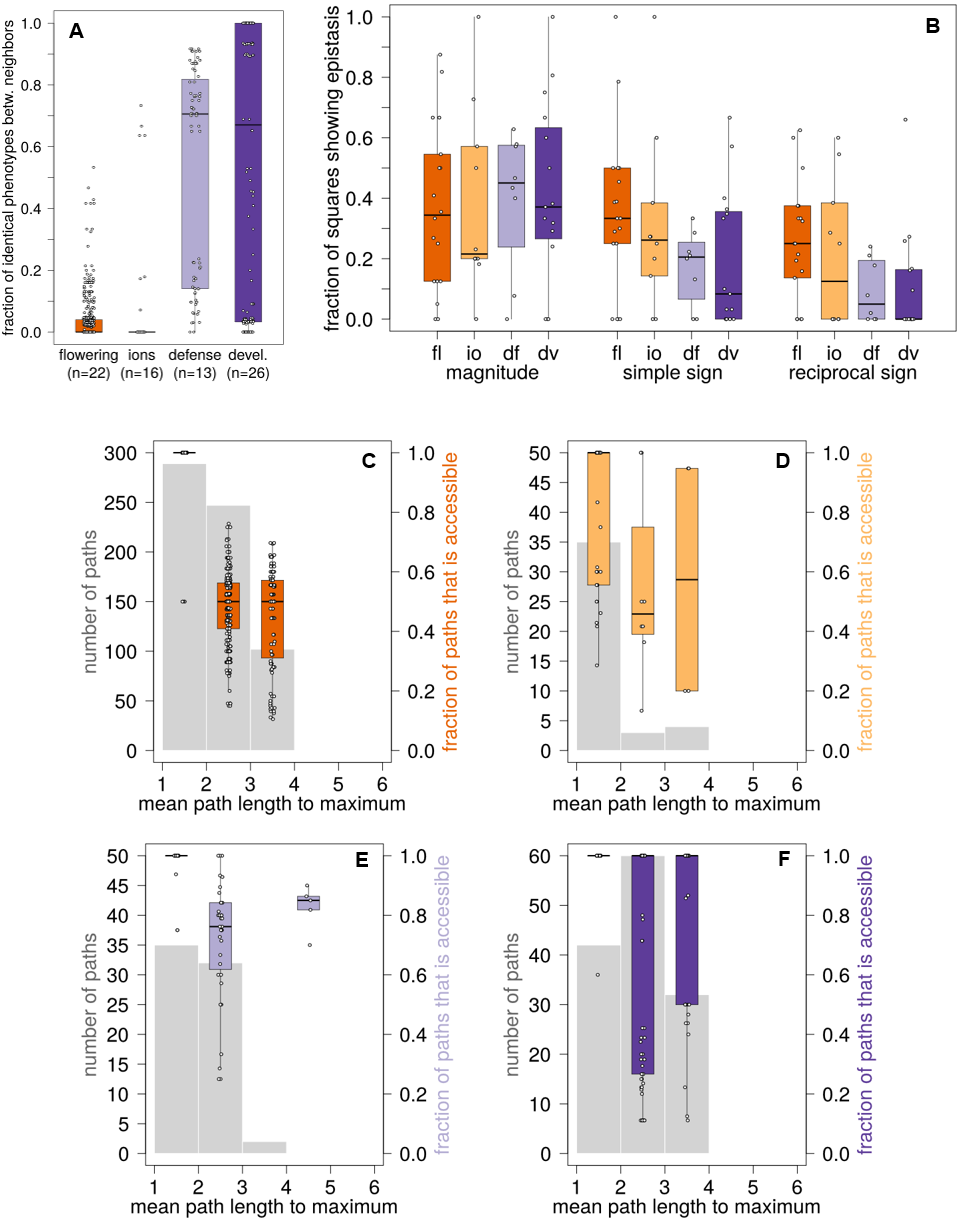

Supplement: S6 Fig — (A) Fraction of genotype network neighbors with identical phenotypic values. We plot the fraction of neighbors with identical phenotypic values (vertical axis) in the form of a box plot for each of the four phenotype categories (“devel.”: development). The number of phenotypes in each category is shown on the horizontal axis as sample size (n). (B) Frequency of different types of epistasis that we obtained when correcting for potential LD. The vertical axis shows the fraction of squares with magnitude, simple sign, and reciprocal sign epistasis as a box plot, where data are grouped according to the type of epistasis and phenotype category (horizontal axis; “fl”: flowering; “io”: ions; “df”: defense; “dv”: development). In each box plot, the central horizontal line indicates the median value, lower and upper box limit shows the values of the first and third quartile, whiskers indicate values within the 1.5-fold interquartile range, and each open circle shows one data point. (C – F) Distribution of mutational path lengths to maximum phenotypic values and fraction of accessible paths with correction for potential LD. For each phenotype, we calculated the mean length of the mutational path from each vertex in the network to the vertex with the maximum phenotypic value. We show the number of paths (left vertical axes) binned according to path length (horizontal axes, path length between 1 and 2 mutational steps, between 2 and 3 mutational steps, etc.) as indicated by the grey histograms. The colored box plot in each bin shows the fraction of paths to the maximum phenotypic value that is accessible (right vertical axes). Each panel shows the result for one phenotype category, namely flowering (C), ions (D), defense (E), and development (F). (TIF) [file pcbi.1008082.s016.tif]

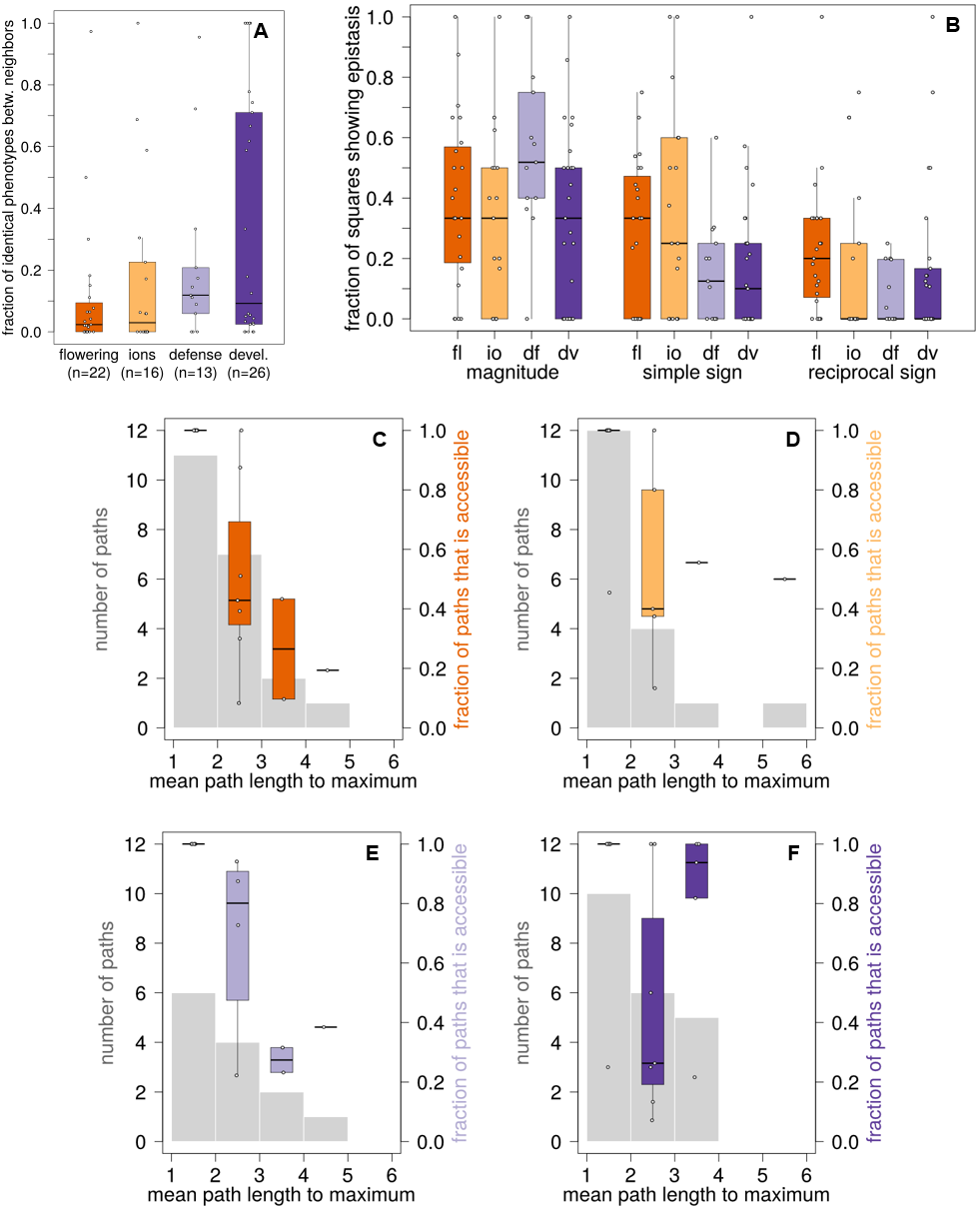

Supplement: S7 Fig — To determine how sensitive our findings of epistasis are to the presence of genomic positions with such alleles, we excluded all loci with such minor alleles from our data set before constructing genotype strings and genotype networks. Data are presented as described for S6 Fig. (TIF) [file pcbi.1008082.s017.tif]

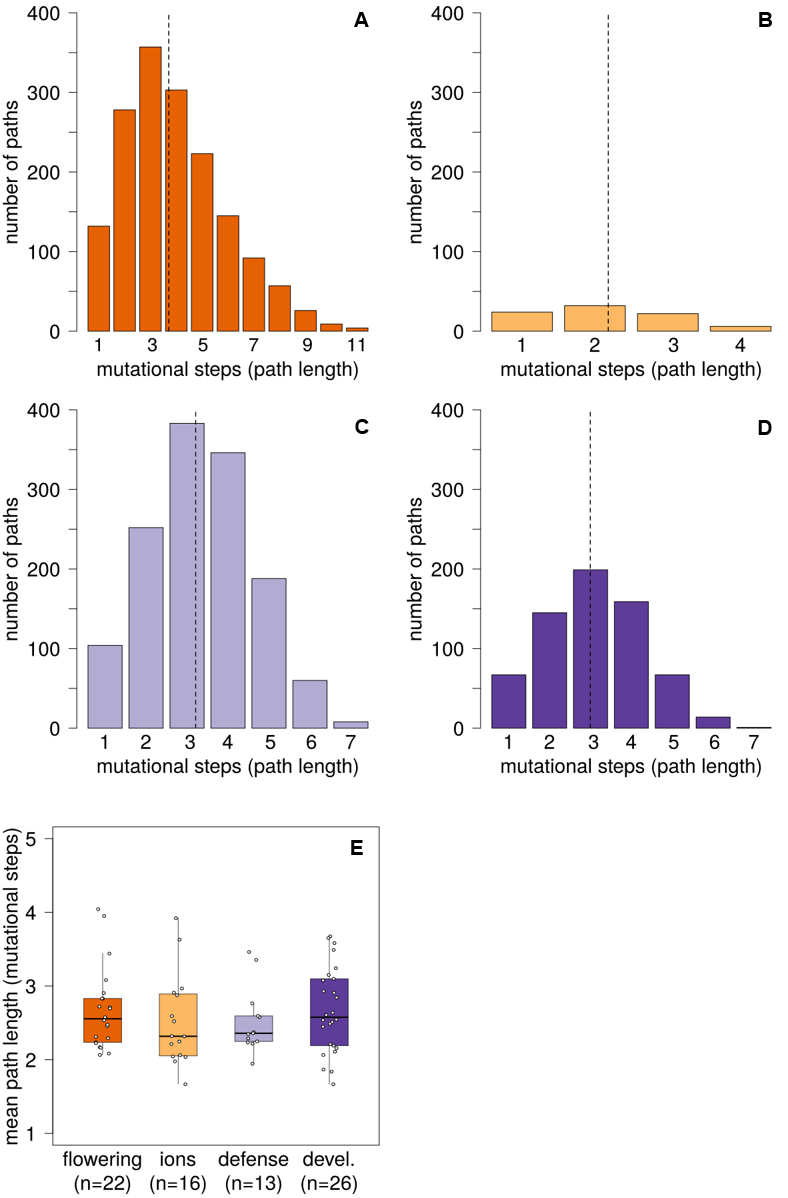

Supplement: S8 Fig — (A-D) Histograms show the distribution of mutational path lengths (horizontal axes) for all mutational paths present in the genotype networks of the four example phenotypes from the main text, i.e., for (A) plant diameter at flowering, (B) arsenic concentration, (C) bacterial growth and (D) plant width. Dashed vertical lines indicate the average mutational path length. (E) Mean mutational path lengths for all phenotypes in each of the four phenotype categories (horizontal axis, “devel.”: development). In each box plot, the central horizontal line indicates the median value, lower and upper box limits show the first and third quartile, whiskers indicate values within the 1.5-fold interquartile range, and each open circle shows one data point. The number of phenotypes in each category is shown as sample size (n). (TIF) [file pcbi.1008082.s018.tif]

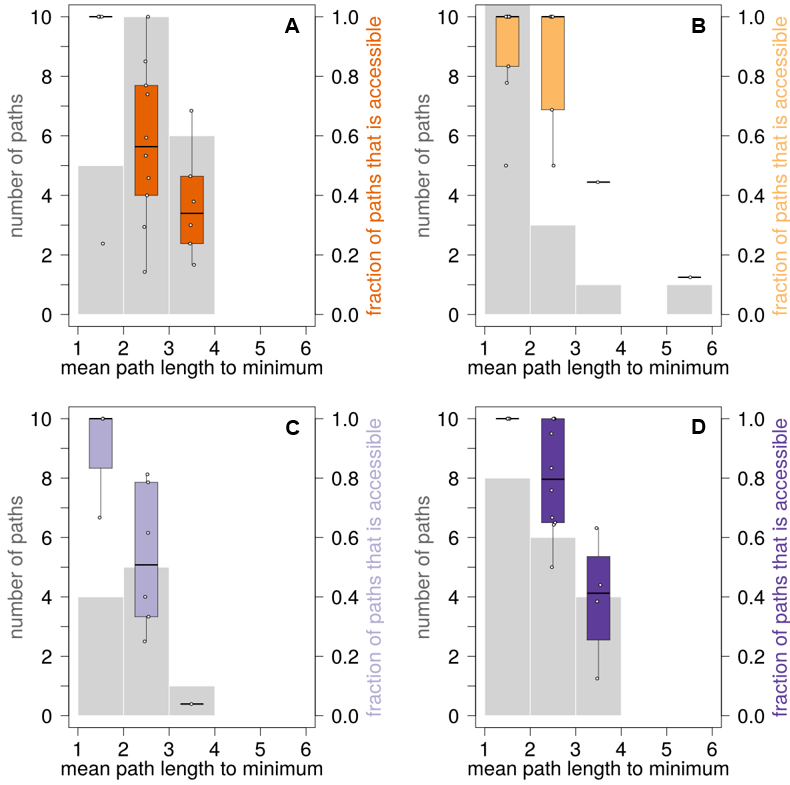

Supplement: S9 Fig — For each phenotype, we calculated the mean length of the mutational path from each vertex in the network to the vertex with the minimum phenotypic value. We show the number of paths (left vertical axes) binned according to path length (horizontal axes, path length between 1 and 2 mutational steps, between 2 and 3 mutational steps, etc.) as indicated by the grey histograms. The colored box plot in each bin shows the fraction of paths to the minimum phenotypic value that is accessible (right vertical axes). Each panel shows the result for one phenotype category, namely flowering (A), ions (B), defense (C), and development (D). (TIF) [file pcbi.1008082.s019.tif]

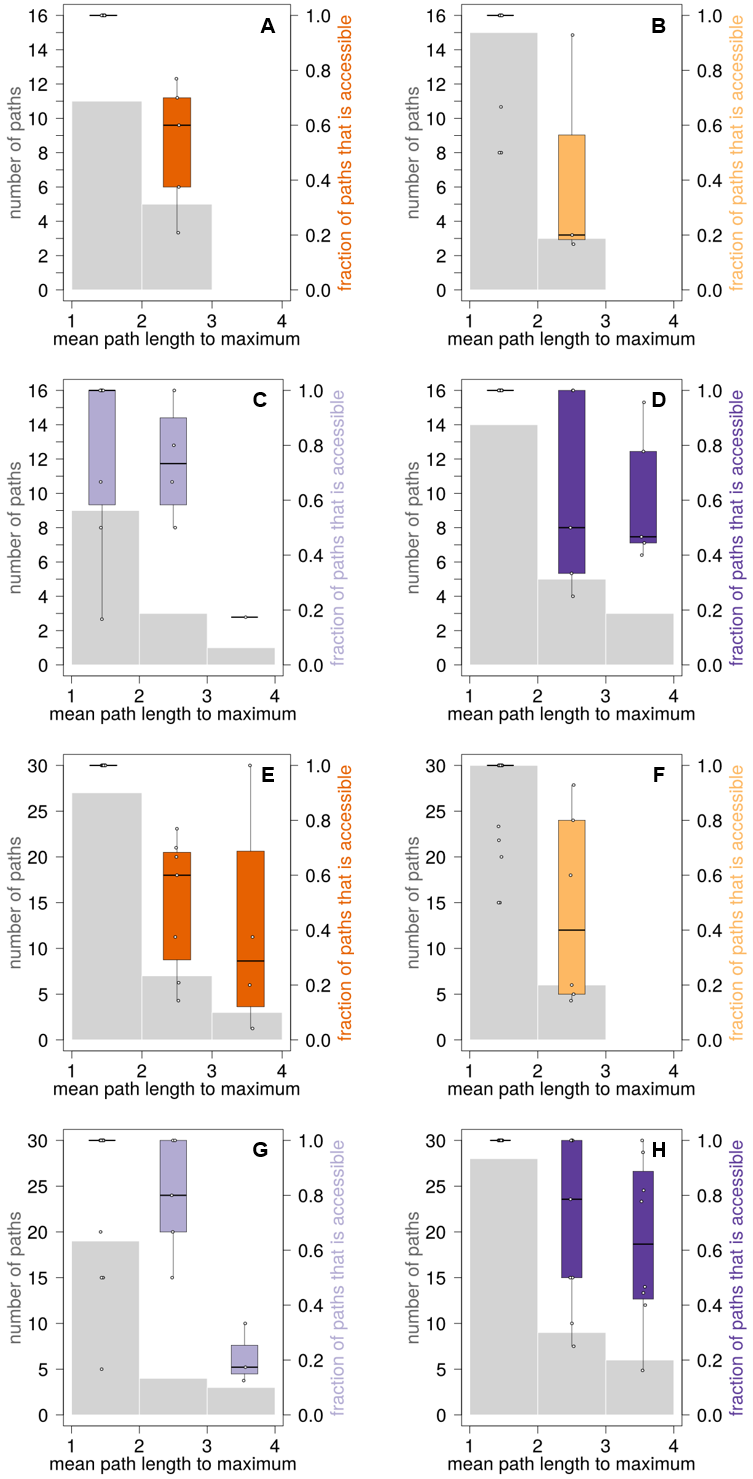

Supplement: S10 Fig — For each phenotype, we reconstructed a genotype network based on P-values whose -log10 score is higher by 0.5 (panels A to D) or lower by 0.5 (panels E to H) than our default P-value for each phenotype. We then calculated the mean length of the mutational path from each vertex in all networks to the vertex with the maximum phenotypic value. We show the number of paths (left vertical axes) binned according to path length (horizontal axes, path length between 1 and 2 mutational steps, between 2 and 3 mutational steps, etc.) as indicated by the grey histograms. The colored box plot in each bin shows the fraction of paths to the maximum phenotypic value that is accessible (right vertical axes). Each panel shows the result for one phenotype category, namely flowering (A and E), ions (B and F), defense (C and G), and development (D and H). (TIF) [file pcbi.1008082.s020.tif]

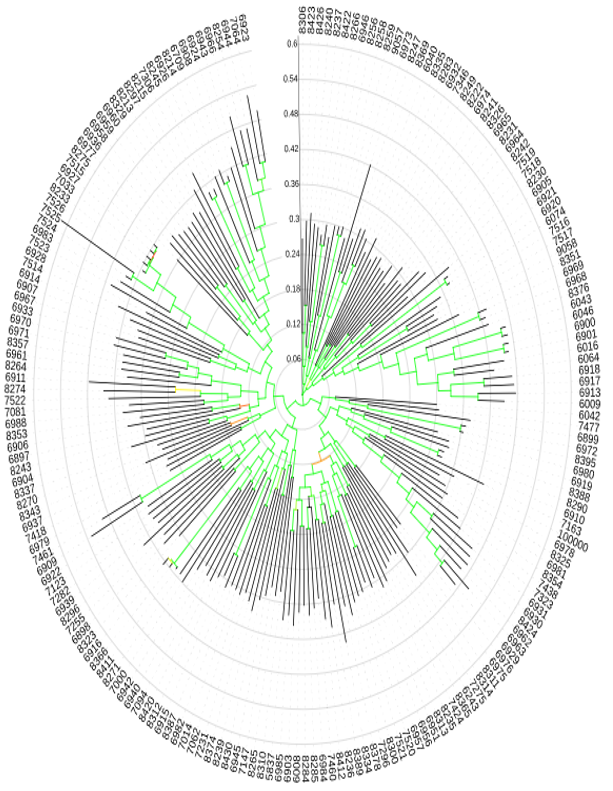

Supplement: S11 Fig — To construct this tree, we first concatenated nucleotides of polymorphic sites in the genomes that were represented on a re-sequencing chip [82]. All such strings had the same length and no position had missing information, that is, the concatenated strings can be aligned without gaps. We used the set of concatenated nucleotide strings as input to FastTree to create a phylogeny with 1,000 bootstraps, and displayed the resulting tree with iTOL. Each leaf corresponds to one accession and leaf labels indicate the accession number. Branches are colored according to their bootstrap support values. Green branches indicate a support value of 1, whereas yellow and orange branches indicate lower support values. Each inner grey circle indicates branch length in steps of 0.06 nucleotide substitutions per site, as indicated by the vertical scale shown in the upper part of the image. (TIF) [file pcbi.1008082.s021.tif]
